# A Multi-Criteria Decision-Making Framework for Fleet Sizing and Replacement Age of Critical Medical Equipment to Support Crisis Resilience: Application to ICU Ventilators

**DOI:** 10.3390/healthcare14213691

**Published:** 2026-10-31

**Authors:** Molk Souguir, Lucas Equeter, Pierre Dehombreux, Frédéric Brichard

**Affiliations:** 1 Machine Design and Production Engineering Lab, Research Institute for the Science and Management of Risks, University of Mons, 7000 Mons, Belgium; pierre.dehombreux@umons.ac.be; 2 Centre Hospitalier de Wallonie Picarde CHwapi, 7500 Tournai, Belgium; frederic.brichard@chwapi.be

**Keywords:** equipment replacement, maintenance, multi-criteria decision making, ventilators, crisis

## Abstract

Background: In health crises, the readiness and availability of equipment are not always achievable. The increasing number and complexity of medical devices require hospitals to establish medical equipment management strategies. In this paper, we define a methodology to help decision-makers select the preferred replacement strategy for critical equipment fleets, such as ICU ventilators, which are life-support equipment whose availability is critical for seriously ill patients. Methods: We present an illustrative case study to identify a preference for a replacement strategy regarding a hospital’s ventilator fleet to ensure the health facility’s routine functioning and resilience to crises. This requires considering multiple factors in a complex environment. In this context, Multi-Criteria Decision Analysis methods are practical tools for assessing each criterion’s impact on the decision process and for objectively evaluating and selecting alternatives. A hybrid Analytic Hierarchy Process (AHP) and additive value model is chosen to perform pairwise comparisons of criteria and evaluate alternatives based on fleet size and replacement period. Results: The results show that it is possible to design a ventilator fleet management strategy that considers the following criteria: service rate in crisis, total cost, staff satisfaction, sensitivity to obsolescence, and ease of procurement and logistics. Among the 12 alternatives, a 9-year replacement period with a 45-unit fleet ranked first, followed by a 15-year policy with the same fleet size. The first rank proved sensitive to the cost weight and the qualitative scores. Conclusions: Effective criteria ranking, score assignment, and the definition of alternatives help develop a consistent decision-making tool that guides decision-makers to the best option for equipment management in normal and critical situations.

## 1. Introduction

Intensive care unit ventilators are a critical asset in healthcare. The strategic management of the ventilator fleet has become a priority for healthcare facilities, particularly after the COVID-19 pandemic, which revealed serious global shortages of respiratory support equipment [1]. Beyond crisis scenarios, ventilators play a major role in critical care, patient support during respiratory failure, surgical procedures, and the management of chronic respiratory conditions. Ventilator availability and reliability affect patient outcomes, clinical and technical staff effectiveness, and the allocation of functional resources. Machine failures can delay treatment, compromise patient safety, and increase the workload of medical and maintenance personnel. However, prematurely replacing a functional ventilator fleet to prevent failure is an inefficient investment that can consume resources meant for other critical healthcare needs. Balancing clinical effectiveness, financial sustainability, and other operational factors creates a multi-dimensional decision challenge in a system that often relies on manufacturer recommendations and health organisations’ guidelines rather than analytical decision frameworks [2].

### 1.1. Critical Medical Equipment

The unavailability or failure of critical medical equipment could cause imminent serious injury or death to the patient. Critical equipment is essential for patient care under normal operating conditions. The criticality of this equipment is not defined by external conditions such as crises and man-made emergencies; it is critical because of the direct relationship between its reliability and the impact on the patient. Critical equipment does not include single-use disposable devices but includes, though is not limited to, the following:

- Ventilators and life-support equipment [3];
- Resuscitation equipment;
- Mission-critical and diagnostic equipment like CT scanners;
- Dialysis machines;
- Sterilisers.

The management of such equipment is therefore addressed over its lifecycle, from acquisition to disposal, rather than at the procurement phase alone. The use phase, during which operating and maintenance resources are used, constitutes the longest stage of that lifecycle [4].

Critical equipment is defined by its impact on patient safety and harm, especially serious injury and possible death, which explains why managing and providing reliable equipment is a priority. Managing this type of equipment requires qualified personnel and a reliable risk assessment for both patient safety and staff well-being. Maintaining critical equipment is a highly sensitive operation because it must be performed in accordance with the manufacturer’s recommendations [5]. In particular, the frequency and methods of preventive maintenance must be followed to ensure equipment availability and readiness after a failure. Maintenance requirements are not to be neglected or altered by the hospital and must be regulated in order to provide an optimal healthcare service that respects the regulations, the manufacturer’s recommendations, and the patient’s right to health and treatment. The hospital can consult experts to optimise the preventive maintenance frequency, but it must always respect the maximum interval suggested by the manufacturer. The hospital is responsible for assessing the PM frequency, repair duration, backup availability, resources, and the overall maintenance and replacement strategy, based on the budget and infrastructure it can provide.

#### ICU Ventilators

An ICU ventilator is a machine that mechanically assists a critically ill patient in breathing. This equipment moves oxygen-enriched air into and out of the lungs by positive pressure through a tube that is placed in the patient’s mouth or nose, or through a tracheostomy [6]. Ventilators push air into the lungs with set amounts of gas and allow passive exhalation depending on several parameters like the respiratory rate, the breath volume, and positive end-expiratory pressure (PEEP).

They require available ICU beds, specialised medical and technical staff, and other essential resources. Due to their invasive use and critical impact on the quality of life and safety of patients, the management and decision making related to ventilators raise serious ethical issues. ICU ventilators combine several factors: clinical criticality, technical complexity, high cost, and a supply chain sensitive to high demand. All this makes them an ideal research object for decision making and equipment management.

### 1.2. Research Objective and Framework

Healthcare facilities face fundamental operational problems: equipment replacement age and fleet size. This decision-making process is often complex for critical care equipment, where multiple conflicting criteria must be considered and where maintenance and investment are interdependent.

Previous research has focused on a maintenance-only approach for multi-criteria applications [7], or single-criterion optimisation of medical equipment management and decision processes, typically the reduction of lifecycle costs [8,9,10] or reliability [11,12].

The application of MCDM methods in healthcare decision-making has addressed problems such as the prioritisation of equipment for maintenance [2,13,14]; prioritisation for replacement [15,16,17]; classification of spare parts and optimisation of inventory management [18]; design of a decision support system for preventive maintenance planning [19]; selection of a maintenance strategy [7]; ranking of factors in order of priority [20]; and hospital performance evaluation [21]. This study introduces MCDM methods for fleet sizing and replacement-age decisions, aiming to evaluate and select an asset management policy that contributes to the hospital’s resilience during a crisis period. The competing alternatives are evaluated across criteria affected by the policy. This helps decision makers choose the most suitable option for all stakeholders (patients, investors, medical staff, technical staff) objectively and comprehensively.

Ventilator replacement decisions require balancing budget constraints against technological obsolescence, equipment reliability against staff satisfaction regarding training burden and equipment user-friendliness, and advancement of clinical features of equipment against the administrative complexity of procurement and logistics. Foremost, compliance with regulatory and quality standards [22] as well as risk management must be guaranteed [23].

Moreover, the literature shows a clear gap regarding the consideration of crises when selecting fleet replacement policies. The ISO definitions of crises emphasise an unstable condition involving significant change, often requiring urgent action to protect life, assets, property, or the environment [24], and situations that exceed the usual means of operation and demand strategic responses [25]. In this context, a health crisis can threaten the patient’s safety, whether it is a public health emergency such as a pandemic or a local health emergency caused by injuries from wars and conflicts, accidents, or locally spreading diseases. An increase in the number of patients is a serious crisis if health facilities do not have the needed capacity to provide health services for critically ill or injured patients. Petanidis et al. [26] raise the same concern. This dimension makes decision making regarding critical equipment a sensitive process since it has a direct impact on the resilience of a hospital to a public, local, or internal health crisis. That is why it has become more important to focus on the implementation of methodologies that support the management of medical equipment [27]. This complex decision requires considering multiple factors and prioritising them objectively. That is why a Multi-Criteria Decision Analysis (MCDA) tool that provides objective judgment, accounts for health stakeholders’ preferences [28], and ranks decision alternatives while considering all conflicting criteria is important for ensuring an efficient decision.

This research aims to provide a multi-criteria decision-making aid for managing hospital medical assets. This approach is designed for the choice of a fleet replacement and sizing policy. Moreover, we emphasise the importance of readiness and resilience in the face of crises. In this context, we suggest different replacement strategies for medical ventilators in terms of replacement periods and spare-equipment policies, under a fixed preventive maintenance policy. They are compared using multi-criteria decision-making (MCDM) methods to select the most suitable decision alternative according to a set of criteria, including patient safety, total cost, and staff satisfaction, among others.

Section 2 presents the methodology as it is intended to be applied in a hospital: the maintenance and replacement policies considered, the AHP weighting of criteria and the additive ranking of alternatives, and the group elicitation of criteria, scores and judgments. Section 3 applies it to an illustrative case study, the ICU ventilator fleet of one hospital, and describes the alternatives, the criteria, the cost model and the simulation. Section 4 presents the results and their sensitivity to the judgments, the qualitative scores and the cost assumptions. Section 5 discusses the results and the limitations, and Section 6 concludes.

## 2. Problem and Methods

### 2.1. Problem Definition

According to ISO 55000:2024, asset management is the coordinated activity of an organisation to realise value from assets [29]. It includes all activities to systematically manage, operate, maintain, and dispose of assets while balancing costs, risk, opportunities and performance. Medical equipment life management is the process of managing the useful life of the equipment from acquisition to disposal. It includes establishing policies for equipment use, maintenance, and replacement planning. This research aims to help decision makers select parameters for an investment and maintenance strategy for critical medical equipment. This includes the selection and evaluation of the critical criteria for the decision, the evaluation and ranking of the different decision alternatives according to these criteria, and the presentation of a support model that guides the decision maker through a multi-criteria decision process.

In the remainder of this section, we cover the methods, beginning with an overview of the maintenance policies considered in this study, followed by the definition of MCDM methods and the description of the decision method.

### 2.2. Maintenance Policies

Maintenance refers to the actions taken to keep equipment, machinery, systems, or software in good working condition and to prevent potential issues that could lead to malfunctions or failures. It involves routine checks, repairs, spare parts replacement, and documentation.

Different types of maintenance can be performed on medical equipment:

- Corrective maintenance is carried out after a fault has been detected and restores the machine to working order, so that it can continue to perform the task required of it.
- Preventive maintenance is carried out at predetermined intervals or after the detection of a deteriorating condition. This type of maintenance, carried out before a failure occurs, significantly reduces the number of failures.
  – Scheduled preventive maintenance is carried out at predetermined intervals.
  – Condition-based preventive maintenance is carried out when a state of deterioration is detected. This is made possible by sensors installed on the machine.
  – Predictive preventive maintenance is carried out according to the evolution of a degradation state.

The maintenance process is defined according to the criticality of equipment [30]. The level of criticality indicates the maintenance strategy that should be assigned to each piece of equipment, as shown in Table 1.

Previous case studies showed a significant reduction in ventilator failures after implementing routine preventive maintenance [31]. This shows that time-based maintenance provides economic benefits by preventing unplanned downtime, extra repair expenses, and replacement costs. Healthcare facilities that implement comprehensive preventive maintenance typically achieve a 20–30% reduction in total maintenance cost. Preventive maintenance extends device lifespan by preventing condition and performance issues from progressing to irreparable damage and failure-induced replacements. Failure to perform preventive maintenance significantly reduces medical equipment lifespan [32]. When performed on schedule, PM helps healthcare facilities keep equipment warranties. Following the manufacturer’s instructions and recommendations for routine preventive maintenance helps extend equipment life and identify potential failure modes before warranties expire.

#### Maintenance Strategy: Sequential PM Policy

**Figure 1:**
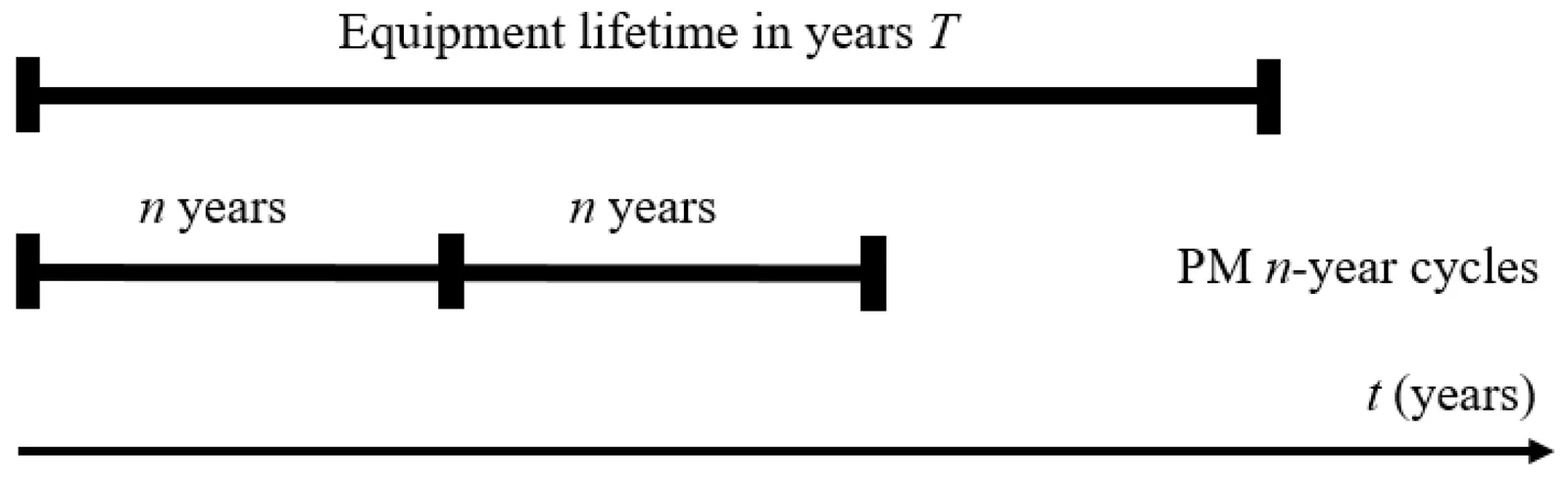
Preventive maintenance *n*-years cycle policy.

**Figure 2:**
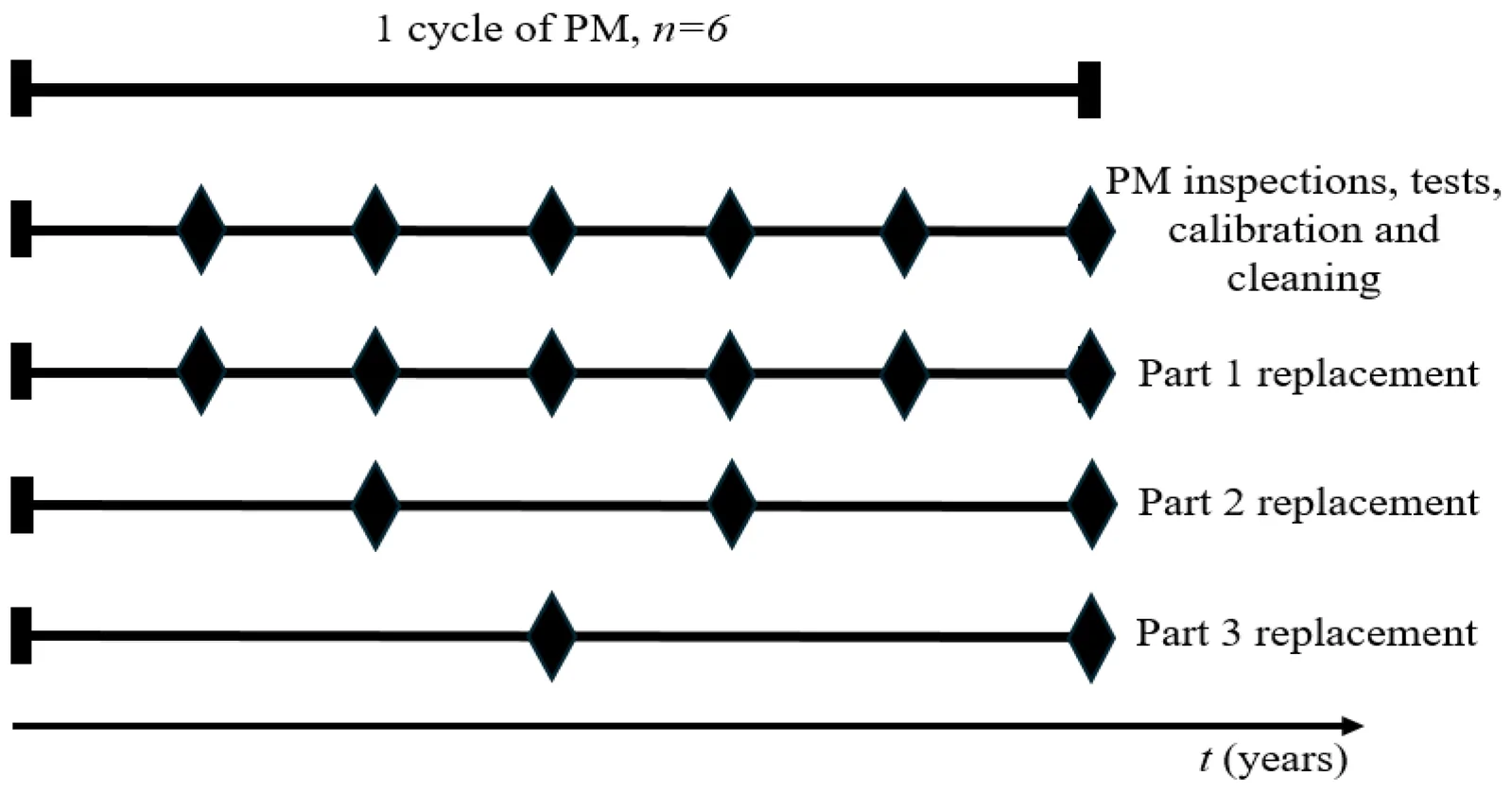
Example of the preventive maintenance *n*-years cycle policy. Part-specific events are shown as diamonds.

We define a sequential PM policy for critical medical equipment (Figure 1). This time-based maintenance strategy plans preventive maintenance across multiple temporal levels: an *n*-year PM cycle that includes recurring annual interventions (Figure 1) and part-specific replacement intervals (Figure 2).

The *n*-year sequential policy, shown in Figure 1, operates on a cycle basis where maintenance occurs at times $T_{0}+I_{p}$, $T_{0}+2I_{p}$,…, $T_{0}+nI_{p}$, where $T_{0}$ is the equipment acquisition year and $I_{p}$ is the PM cycle interval (years), and the PM cycle resets at $T_{0}+\left(n+1\right)I_{p}$.

For a fleet of *N* units with acquisition years $T_{0}^{\left(1\right)},T_{0}^{\left(2\right)},\ldots ,T_{0}^{\left(N\right)}$, the PM intervention schedule for unit *i* over one cycle is:

(1) $$S^{\left(i\right)}=\left\{T_{0}^{\left(i\right)}+j\cdot I_{p}\;|\;j=1,2,\ldots ,n\right\}$$

where *n* is the number of PM steps per cycle and $I_{p}$ is the PM cycle interval (years).

Determining the appropriate cycle interval depends on several factors: the manufacturer’s recommendations; warranty requirements; standards and regulatory requirements; maintenance records; failure rates for specific parts; equipment criticality; and budget capacity and resource constraints.

This complex strategy introduces differentiated scheduling for each part of the equipment, along with annual inspections and testing, all included in one PM cycle that restarts after n years until the equipment is replaced. Despite the complexity of managing sub-cycles, this strategy, once defined, becomes more convenient for the maintenance department because it follows a schedule described by a time-estimation table that outlines the PM activity for each year of the cycle for each piece of equipment.

### 2.3. Age-Based Replacement Policy

An age-based replacement policy defines a replacement strategy for the equipment fleet. Under this policy, an asset is replaced at a predetermined age *T*. This policy is a fundamental preventive maintenance strategy because it relies on replacing equipment before catastrophic failures. An age-based replacement policy can also be failure-related, which means replacing the asset upon failure if it occurs before the predetermined age.

To determine the optimal replacement time, we rely on two main factors: the manufacturer’s end-of-life limit and the total cost of ownership, which includes PM and CM costs. This requires balancing the purchase opportunity cost, the cost of preventive activities, and the cost of corrective replacements.

### 2.4. MCDM Methods

Multi-Criteria Decision Analysis (MCDA) is a tool set designed to aid decision makers in evaluating and prioritising multiple, often conflicting, criteria. In complex decision-making scenarios, such as policy development, project selection, or resource allocation, MCDA provides a structured approach to assess various alternatives based on a range of quantitative and qualitative factors.

Multi-criteria decision support is the activity of using models to answer stakeholder questions in a decision-making process [33]. These models make it possible to clarify an answer to the problem posed, and in most cases lead to the recommendation or promotion of a solution that increases coherence between the evolution of the process, the stakeholder’s objectives and value system.

**The decision maker** is the person who must make a decision from among several options. In collaboration with the experts, they determine the relevant criteria, as well as the weighting of these criteria.

**The expert** is the person mandated by the decision maker to recommend an option from among those presented. Their job is to implement a decision aid.

**The alternatives** are defined in the decision-aiding process. A choice is made among these.

**The criterion** is an element used to evaluate each option. Criteria are ranked, and their weights measure the relative importance of each criterion.

Applying MCDM methods to make the right decision regarding the management of medical ventilators involves the following steps:

1. Identify the asset management alternatives;
2. Identify all the relevant criteria and sub-criteria for criticality assessment of devices;
3. Determine weighting values for all criteria and sub-criteria;
4. Set up grades and determine intensities for each criterion;
5. Evaluate alternatives with respect to each criterion and assign grades and scores;
6. Identify the appropriate alternative.

### 2.5. AHP Method for Criteria Weights Determination

**Figure 3:**
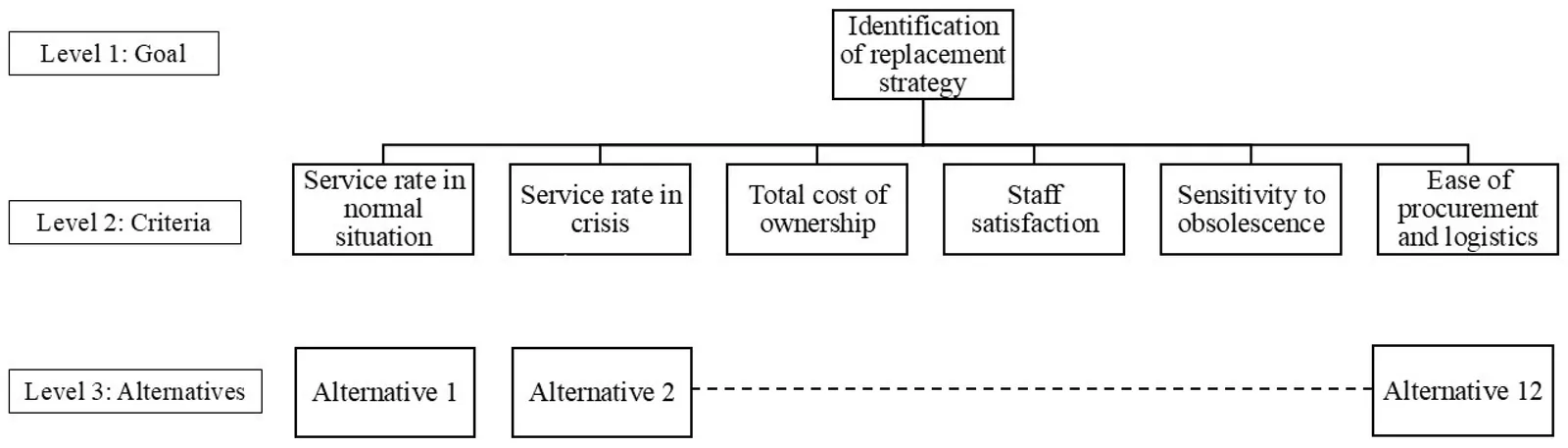
AHP Hierarchy structure.

The Analytic Hierarchy Process (AHP) is a structured method developed by T.L. Saaty [34] to organise and analyse complex decisions using mathematics and personal preferences. AHP allows decision makers to model a complex problem in a hierarchical structure [35] through the relationships among the goal, criteria, sub-criteria (if any), and alternatives (Figure 3).

The Analytic Hierarchy Process method was selected for this analysis due to its ability to accommodate both qualitative and quantitative criteria, structure complex multi-criteria problems, and generate consistency measures to validate the decision assessments. It is necessary to construct the pairwise comparison matrices. These pairwise comparisons are made to establish priorities among the criteria. These comparisons are made using a Saaty scale of 1 to 9 (provided in Table 2), where 1 indicates equal importance and 9 indicates extreme importance of one element over another [36].

In order to find the best weights using this method, a matrix of pairwise ratios between each pair of criteria is introduced. All elements of the matrix are non-null and positive and diagonal elements are always equal to 1, as a criterion compared to itself is equally important.

For $n_{c}$ criteria, the pairwise comparison matrix ***A*** is an $n_{c}\times n_{c}$ matrix where $a_{ij}$ represents the relative importance (Saaty intensity of importance) of criterion *i* over criterion *j*.

(2) $$A=\left[\begin{array}{cccc} 1 & a_{12} & \ldots & a_{1n_{c}}\\ \frac{1}{a_{12}} & 1 & \ldots & a_{2n_{c}}\\ \vdots & \vdots & \ddots & \vdots \\ \frac{1}{a_{1n_{c}}} & \frac{1}{a_{2n_{c}}} & \ldots & 1 \end{array}\right]$$

The priority vector s is obtained by Saaty’s additive normalisation method [34], in which each column of *A* is divided by its sum and the resulting rows are averaged:

(3) $$s_{i}=\frac{1}{n_{c}}\sum_{j=1}^{n_{c}}\frac{a_{ij}}{\sum_{k=1}^{n_{c}}a_{kj}},\;i=1,\ldots ,n_{c}$$

so that the elements of *s* sum to one. This procedure, proposed by Saaty as an approximation to the principal eigenvector, coincides with it when *A* is consistent.

The maximum eigenvalue used in the consistency check is estimated with these weights as

(4) $$\lambda_{\max}=\frac{1}{n_{c}}\sum_{i=1}^{n_{c}}\frac{1}{s_{i}}\sum_{j=1}^{n_{c}}a_{ij}\;s_{j}$$

The consistency of the criteria pairwise comparisons (matrix *A*) is checked using the consistency index (CI) and the consistency ratio (CR).

(5) $$\mathrm{CI}=\frac{\lambda_{\max}-n_{c}}{n_{c}-1}$$

The consistency ratio is calculated as follows.

(6) $$\mathrm{CR}=\frac{CI}{RI}$$

where *RI* is the random consistency index, which depends on the number of criteria $n_{c}$. If CR<0.1, the consistency is acceptable; otherwise, the pairwise comparisons need to be revised.

### 2.6. Weighted Sum for Alternatives Ranking

Once the criterion weights $w_{j}$ are obtained from the pairwise comparison matrix *A* above, the score of each alternative on each criterion is not derived from a further round of pairwise comparison between alternatives. Instead, let $x_{i}^{\left(j\right)}$ denote the value alternative *i* achieves on criterion *j* (the simulated availability or cost figure, or the analyst-assigned qualitative rating). This value is converted into a normalised score ${\hat{s}}_{i}^{\left(j\right)}$ (between 0 and 1) by comparing it with the best value reached by any alternative on that criterion.

For a criterion to be maximised:

(7) $${\hat{s}}_{i}^{\left(j\right)}=\frac{x_{i}^{\left(j\right)}}{\max_{k}x_{k}^{\left(j\right)}}$$

and for a criterion to be minimised:

(8) $${\hat{s}}_{i}^{\left(j\right)}=\frac{\min_{k}x_{k}^{\left(j\right)}}{x_{i}^{\left(j\right)}}$$

The overall priority (final score) of alternative *i* is then the weighted sum of its normalised scores across all $n_{c}$ criteria:

(9) $$p_{i}=\sum_{j=1}^{n_{c}}{\hat{s}}_{i}^{\left(j\right)}\cdot w_{j}$$

Alternatives are ranked by sorting these final scores in descending order. The additive aggregation is the synthesis rule of the AHP itself [36]; therefore, the weights obtained from pairwise comparisons keep their meaning as trade-off coefficients between criteria. This approach assumes full compensation between criteria and makes each final score linear in the weights, which makes it possible to compute the thresholds that would alter the ranking.

#### 2.6.1. Criteria Weight Determination

Criteria weights are determined using the Analytic Hierarchy Process (AHP) pairwise comparison procedure [34]. For each pair of criteria, decision makers should judge the relative importance of one criterion over the other using Saaty’s 1–9 intensity scale, where 1 indicates equal importance, and 9 indicates extreme importance of one criterion over the other; reciprocal values (1/3, 1/5, …, 1/9) are assigned to the inverse comparisons. These judgments are assembled into a square pairwise comparison matrix.

Criteria weights are derived from this matrix, which yields the relative priority (weight) of each criterion. They are obtained using Saaty’s approximate method: each column of the pairwise comparison matrix is normalised to sum to one, and the weight of each criterion is the average of its row. The consistency of the judgments is verified by computing the consistency ratio (CR), with CR < 0.10 taken as the threshold for acceptable consistency.

The resulting weights are subsequently applied to the normalised performance scores of each alternative across all criteria to obtain the final weighted ranking.

#### 2.6.2. Decision Criteria

Managing and classifying medical devices and spare parts while focusing on optimising maintenance and inventory management requires various criteria. The criteria for classification include economic, operational, and risk factors, alongside specific measures like the age of equipment, failure rates, and service support. For instance, Teixeira [18] emphasises the function, production impact, and lead time of spare parts for inventory management. Jamshidi [2] introduces criteria such as mean time between failures and economic loss to prioritise medical device maintenance. Additionally, Albadr [37] discusses criteria such as equipment age and physical risk to improve decision making in maintenance practices. Also, Carnero [38] highlights the importance of economic and operational criteria in developing effective maintenance policies. These comprehensive frameworks ensure that the selection of criteria for maintenance and inventory decisions contributes to reducing downtime, guaranteeing optimal health services, and enhancing overall efficiency.

The criteria, their importance, and the scoring are elicited from managers of different hospital departments. The criteria for comparison are drawn from the hospital’s designated decision-making committee, which may include medical, financial, biomedical, and IT management. This composition may vary from one hospital to another but should reflect the body that, in the hospital where this methodology would be deployed, actually approves investment and replacement plans for critical medical equipment. Participants can develop criteria-related parameters during decision sessions moderated by the decision-aiding expert, where each participant can validate or suggest decision criteria, express their individual judgment on qualitative criteria according to the presented evaluation grids, and then agree on an overall score. This group elicitation design follows the practice reported by Bai et al. [39], who structured the assessment of hospital resilience determinants through moderated group decision-making sessions with a panel of hospital experts.

The biomedical ethics committee then has the responsibility, as a first step, to review and validate the selection and scoring of criteria, and, as a second step, to review the final ranking of alternatives and verify that a management strategy guarantees sufficient equipment capacity. It should be entitled to reject any alternative that does not meet its conditions or that falls below a safety threshold.

## 3. Illustrative Case Study: ICU Ventilators with Crisis Awareness

This section applies the methodology of Section 2 to the ICU ventilator fleet of one hospital. The case study is illustrative and departs from Section 2 in one respect: the criteria, the qualitative scores and the pairwise comparisons were set by the authors and validated by the hospital’s biomedical engineer responsible for the fleet, instead of being elicited from the hospital’s decision committee. The fleet composition, the decision alternatives, indicative labour rates, indicative unit prices, PM durations and the crisis capacity come from the hospital, and the reliability parameters are indicative values (Section 3.5). The case study therefore demonstrates the chain from simulation to ranking, not the elicitation protocol.

In this case study, a hospital has 36 ICU beds, each connected to an ICU ventilator. It operates a total fleet of N=45 ICU ventilators, of which $N_{use}=36$ are in service and $N_{sb}=9$ are on standby in a ready-to-use state (Table 3).

(10) $$N=N_{use}+N_{sb},$$

Each ventilator operates for 24 h per day and each patient uses the same ventilator for the entire duration of their stay in intensive care, except in the event of a breakdown. Therefore, no hourly sampling is assumed to be required for ventilators.

ICU bed availability is tied to ventilator availability. A bed is only available if it is connected to a functional ventilator. The initial ICU ventilator fleet contains equipment of two different ages (Table 4). The 20 newer ventilators are in service, along with 16 ventilators from the older fleet segment.

Daily clinical demand requires the availability of ICU ventilators, leaving limited time for cleaning, checks, inspections, and corrective and preventive maintenance of ventilators in service.

During periods of crisis (an increase in the number of patients), it is essential to have more ventilators ready for use. This requires a maintenance and replacement policy that ensures rapid recommissioning of out-of-service devices and the availability of reliable ventilators.

We define the requirements for ICU beds in the event of a healthcare crisis, by analogy with the COVID-19 period. The hospital was able to expand the ICU capacity from 36 beds under normal conditions to 44 beds, a 22% increase in the number of patients, thanks to 15 extra ventilators retained from the previous replacement, which had been in service for 15 years (Table 5).

### 3.1. Decision Alternatives

Decision alternatives for a maintenance and replacement policy for the ICU ventilator fleet are proposed based on their replacement periods, preventive maintenance policies, and in-stock equipment. These parameters affect the number of ventilators available for immediate use, which can affect the hospital’s capacity and response time in a crisis, and more specifically, in case of an increase in the number of patients in intensive care.

The decision alternatives, in this application, were suggested by the hospital maintenance manager, and the maximum replacement age and total fleet size were bounded by the manufacturer’s recommendations and by the hospital’s investment cycle. This approach is consistent with WHO guidance on decommissioning, which recommends basing the withdrawal of a medical device on its performance and cost history, a device being due for replacement once its performance begins to decline [40].

For all ventilator management policy options, one preventive maintenance intervention is proposed per year.

The decision alternatives, provided in Table 6, vary in terms of replacement period and in-stock equipment. The hospital requires ICU ventilators to be available and reliable for patients occupying the 36 ICU beds, and to be ready in case of a sudden increase in patients, which represents a crisis. The hospital’s current human resources can handle only a 22% increase in ICU patients, but it must still have equipment ready for this increase, as the first responder to a crisis in its area, before it can redirect patients and monitor the situation.

The philosophy is to balance equipment availability, ownership cost, and other decision-making criteria to select the best alternative that ensures patient safety, stays within the hospital’s budget, and satisfies patients and medical and technical staff.

The suggested equipment replacement years affect purchase cost because they change the number and timing of replacements, which determines the price indexation applied and maintenance cost over the next 15 years, since electromechanical systems tend to have an increasing failure rate with age and use.

### 3.2. Decision Criteria

In this application, six criteria are defined, and used to rank the suggested equipment management decision alternatives:

- Service rate in normal operation;
- Service rate during a crisis;
- Total cost of ownership;
- Staff satisfaction;
- Sensitivity to obsolescence;
- Ease of procurement and logistics.

Some criteria are evaluated qualitatively using defined grids, while others are evaluated quantitatively through specific calculations.

In this application, which serves an illustrative purpose, the criteria selection and score assignment were performed by the authors. The qualitative criteria grids were suggested based on an example purchase-decision evaluation grid provided by the hospital. In this case, the hospital’s biomedical engineer responsible for equipment fleet management validated the criteria, their scores, and the pairwise comparisons. As explained in Section 4, the service rate in normal operation did not allow discriminating between alternatives and was not kept.

#### 3.2.1. Qualitative Criteria

These criteria are evaluated using grids with ordinal scales. The scores are usually assigned by experts or decision makers.

- Staff satisfaction

This criterion describes how satisfied the medical, paramedical, and technical staff are with the equipment available for use. This is measured by the ease of use and management of the equipment according to staff preferences, as described in Table 7.

- Sensitivity to obsolescence

This criterion describes how modern the equipment’s features are. It describes the effectiveness of the clinical and technological features in treating the patient and in the medical staff’s use of the equipment, as described in Table 8.

- Ease of procurement and logistics criterion

This criterion describes the purchase process, equipment acquisition logistics, and maintenance planning management, as described in Table 9.

#### 3.2.2. Assignment of Qualitative Scores

After defining the scales and indicators of each criterion, scores were assigned to criteria according to a structured judgment of the decision alternative under consideration. These scores indicate each suggested strategy’s performance, whether as a calculated cost or as a qualitative description that places the alternative at a certain position on the criterion scale.

This step follows a defined process:

1. Review the grid for the relevant criterion;
2. Assess the alternative’s performance according to the evaluation grid;
3. Match the alternative’s characteristics to the level of the criterion that best fits its performance;
4. Assign the corresponding score from the criterion’s grid to the alternative.

This step represents the decision maker’s and the decision expert’s personal input to the model. Assigning scores to each criterion requires expert judgment, the availability of technical specifications, and institutional data sufficient to evaluate the criteria according to each decision alternative. For example, for the staff satisfaction criterion, the 7-year replacement period alternatives were assigned a score of 7.5, since newer equipment might align with staff preferences, but frequent changes can be inconvenient for those who prefer familiar technology. For the sensitivity to obsolescence criterion, the 9-year replacement period alternatives were assigned a score of 10: a balanced fleet of equipment, of adequate age without excessive change, which might match the medical staff’s preferences and require less effort from the logistics and technical staff. For the ease of procurement and logistics criterion, the 12-year replacement period alternatives were assigned a score of 10: this longer interval means less frequent procurement and acquisition logistics.

#### 3.2.3. Quantitative Criteria

These criteria are scored and expressed on numerical scales, such as cost in euros or service rate in percentages.

- Service rate

This research aims to help decision makers choose the most suitable replacement strategy to ensure equipment readiness for daily use and availability in case of an increase in the number of patients, thereby improving the hospital’s resilience during such a crisis.

The service rate α:

(11) $$\alpha =\frac{N_{av}}{N_{need}}$$

The service rate α is a patient safety indicator, which, in this case, is evaluated based on the availability of reliable equipment for each patient within the hospital’s capacity. If α<1, the number of available pieces of equipment is less than the number needed, which can endanger patient safety and lead to treatment delays and potentially serious health consequences.

The service rate under normal conditions is assessed against a threshold of 36 ventilators, which is the number of ICU beds in the hospital:

(12) $$\alpha =\frac{N_{av}}{36}$$

The service rate during a crisis is assessed against a threshold of 44, which is the previously proven maximum capacity of the hospital:

(13) $$\alpha =\frac{N_{av}}{44}$$

- Total cost of ownership

The cost of ownership is the total cost of owning an asset during its useful life. It includes the cost of purchasing, implementing, maintaining, and replacing equipment. In this research, we consider only the purchase cost and the preventive and corrective maintenance costs, because in most medical equipment acquisition cases the implementation and disposal costs are included in the purchase price.

(14) $$C=C_{v}+C_{m}$$

(15) $$C=C_{v}+C_{c}+C_{p}$$

### 3.3. Cost Model

- Purchase cost

To calculate the purchase cost of ICU ventilators for each suggested alternative over a period of 15 years, it is important to state the initial age of each equipment group and the resulting number of replacements per replacement period. This affects the purchase cost through the number of units that must be bought over the next 15 years and the price indexation, which depends on the year of purchase.

(16) $$c_{v}=25,000\;€,$$

(17) r=0.02,

(18) $$C_{v}=\sum_{i=1}^{k}c_{v}\cdot {\left(1+r\right)}^{y_{i}}\cdot N_{repl,i}$$

For *T* the replacement period, *a* the current machine age, and *H* the planning horizon, the replacement years, relative to now, form the sequence.

$$\delta =\left\{\begin{array}{cc} 0, & \mathrm{if}\;a\geq T,\\ T-a, & \mathrm{if}\;a<T. \end{array}\right.$$

$$t_{n}=\delta +\left(u-1\right)T,\;u=1,\ldots ,z,\;z=\left\lceil \frac{H-\delta }{T}\right\rceil .$$

$$Y=\left\{\;\delta ,\;\delta +T,\;\delta +2T,\;\ldots ,\;\delta +(z-1)T\;\right\}.$$

If a≥T, then δ=0, z=⌈H/T⌉ and Y={0,T,…,(z−1)T}, that is, an immediate first replacement.

A replacement falling exactly at the end of the planning horizon (y=H) is not counted: a unit bought at *H* would provide no service within the horizon.

If a<T, then δ=T−a, the first replacement is in T−a years, followed by one every *T* years until *H*.

For alternative 1, the replacement period *T* is set at 7 years, with 2 pieces of equipment in stock. The fleet of equipment that is 15 years old (25 ventilators) must be renewed immediately; however, 7 ventilators are decommissioned and only 18 new units are purchased. This group of equipment will therefore be purchased three times over the next 15 years.

For the 20 ventilators that are 6 years old, the first replacement will occur in 1 year, and a further replacement will occur in year 8; the replacement normally due in year 15 falls at the limit of the planning horizon and is not counted.

All costs are expressed in nominal euros: purchase prices follow the hospital’s framework contracts, indexed at r = 2% per year, and costs are not discounted. However, a finite horizon truncates the replacement cycles differently for each replacement age. To circumvent this limitation, the alternatives can also be compared on the basis of an equivalent annual cost (EAC) at constant prices. The EAC treats each policy as a repeating cycle and requires no assumption about the residual value of the fleet at H.

(19) $$\mathrm{EAC}=\frac{Nc_{v}}{T}+\frac{C_{m}}{H}$$

However, the EAC approach introduces limitations of its own, as it disregards the initial age of the ventilator fleet. Therefore, it is more representative of a steady state than of a transient state, or a one-off decision accounting for a pre-existing situation.

- Preventive maintenance cost

Because ICU ventilators are critical equipment, the hospital follows a time-based maintenance process to ensure device reliability, in line with the manufacturer’s recommendations and safety standards. Because patients depend on these respiratory support devices, equipment failure poses a critical safety risk and requires preventive measures.

These PM activities for ventilators include cleaning and disinfection; performance verification and quality assurance, such as visual inspection; electrical safety tests; calibration; alarm verification; functional testing; and parts replacement according to their expected lifespan.

In this case, preventive maintenance of the ICU ventilators is carried out over a 6-year cycle (Table 10).

The preventive maintenance cost is the sum of the yearly PM costs for each year of the cycle, for each item of equipment.

(20) $$C_{p}=k\cdot N\cdot \frac{T}{6}\sum_{i=1}^{6}C_{p_{i}}$$

(21) $$C_{p_{i}}=C_{l_{i}}+c_{sp_{i}}$$

- Corrective maintenance cost

Corrective maintenance is an important process for ventilator management. The critical environment and consequences require immediate, effective intervention to restore equipment functionality after failures. Delays in corrective maintenance increase the risk of cumulative breakdowns. This increases downtime costs, affects repair quality, causes secondary damage to related equipment parts, and creates a need for emergency equipment rental or spare-parts orders that cost more than routine maintenance and supply-chain costs. The cost of corrective maintenance includes, at each failure event, the cost of spare parts and labour. The information on the costs and downtime is provided in Table 11. Yoshioka et al. [31] conducted a seven-year study of mechanical ventilators and found that batteries and sensors were the most frequently replaced parts, with their failures accounting for most cases. Therefore, to simplify the simulation, we simulate only these two failure modes for corrective maintenance cost calculations, and we do not consider system failures (leaks, wear, and so on).

(22) $$C_{c}=\sum_{i=1}^{n_{fpart}}\left(q_{i}\cdot c_{sp_{i}}+C_{l_{i}}\right)$$

### 3.4. Simulation

First, to simulate and track the equipment activity, the statuses of the ventilators are defined as in Table 12.

These statuses allow defining the events that can change equipment status and, in turn, simulate failures, corrective maintenance, preventive maintenance, and availability. Table 13 lists the significant events that can change ventilator status.

The failure event is triggered by an occurrence of a failure (the inter-event duration following a Weibull distribution). This automatically sends the equipment to repair and starts the “Back to service” event that replaces the defective equipment with a standby unit if the availability of spare equipment is verified. If none is available, the defective device is replaced as soon as it, or another device, has been repaired. The preventive maintenance event occurs once a year for each device during a predefined period, according to a 6-year cycle.

### 3.5. Reliability Modelling for Corrective Maintenance

Reliability is a device characteristic measured by the probability that it will perform a required function under given conditions for a given period of time.

The objective is to estimate the expected cost of corrective maintenance for each suggested decision alternative by estimating the number of ventilator failures over the 15-year planning horizon.

- Maintenance assumptions
  – The inflation rate is not considered in the spare parts cost;
  – Corrective repair or a preventive maintenance intervention for a specific part (part replacement) sets the part to an as good as new state;
  – Each ventilator is modelled according to the preventive maintenance strategy of the year of repair or the strategy-driven replacement period;
  – The failure modes are independent;
  – Maintenance history is kept confidential; realistic parameters are used to estimate failures and calculate corrective maintenance costs.

- Weibull parameters and equations

Time-to-failure for each component was modelled using a two-parameter Weibull distribution [41], with shape and scale parameters β,η>0 given in Table 14. At each replacement or repair event, the component was assumed to return to an “as good as new” condition, and a new failure time was drawn from the Weibull distribution conditional on this reset.

The scale parameter η relates to the mean time to failure (MTTF) via

(23) $$\eta =\frac{\mathrm{MTTF}}{\Gamma \;\left(1+\frac{1}{\beta }\right)}$$

and the hazard function is

(24) $$h\left(t\right)=\frac{\beta }{\eta }{\left(\frac{t}{\eta }\right)}^{\beta -1}$$

Given a component that has already survived to age $t_{a}$, the cumulative hazard accrued up to that age is $H\left(t_{a}\right)={\left(t_{a}/\eta \right)}^{\beta }$. The time of the next failure, $t_{\mathrm{next}}$, was obtained by inverse-CDF sampling of the residual life conditional on $t_{a}$:

(25) $$t_{\mathrm{next}}=\eta {\left[H\left(t_{a}\right)-\ln\left(1-u\right)\right]}^{1/\beta },\;u∼\mathrm{Uniform}\left(0,1\right)$$

so that the residual time to failure is $t_{\mathrm{next}}-t_{a}$. This formulation ensures that components which have already accumulated wear are not erroneously resampled as if they were new.

The choice to simulate these two parts for this application is based on the hospital’s CMMS history, as they represent the most frequent failures, and an FMECA conducted by the hospital that identified these failure modes as the most critical. The raw maintenance data is confidential, and reliability parameter figures are of the same order of magnitude as the recorded ones, as verified by the maintenance engineer. This approach follows established practice in device reliability and risk assessment, where failure data is limited or restricted and parameter estimates must combine available maintenance records with structured expert judgment [42].

Because only sensor and battery failures are simulated, the corrective maintenance cost and downtime reported here are lower bounds, and the simulated service rates are upper bounds. The Weibull parameters in Table 14 are indicative values of the same order of magnitude as the hospital’s records, not estimates fitted to them.

- Monte Carlo simulation

The failure rate of electromechanical systems varies over time due to wear or ageing. In each Monte Carlo iteration, for each unit, the planning horizon was simulated taking into consideration the preventive maintenance schedule and the failure events for the battery and sensor as they occurred over the 15-year horizon, with each failure time drawn using Equation (25).

Results are aggregated across all iterations to obtain the mean number of failures for each part (battery and sensor). The corrective maintenance cost is calculated for the equipment fleet for each decision alternative.

**Figure 4:**
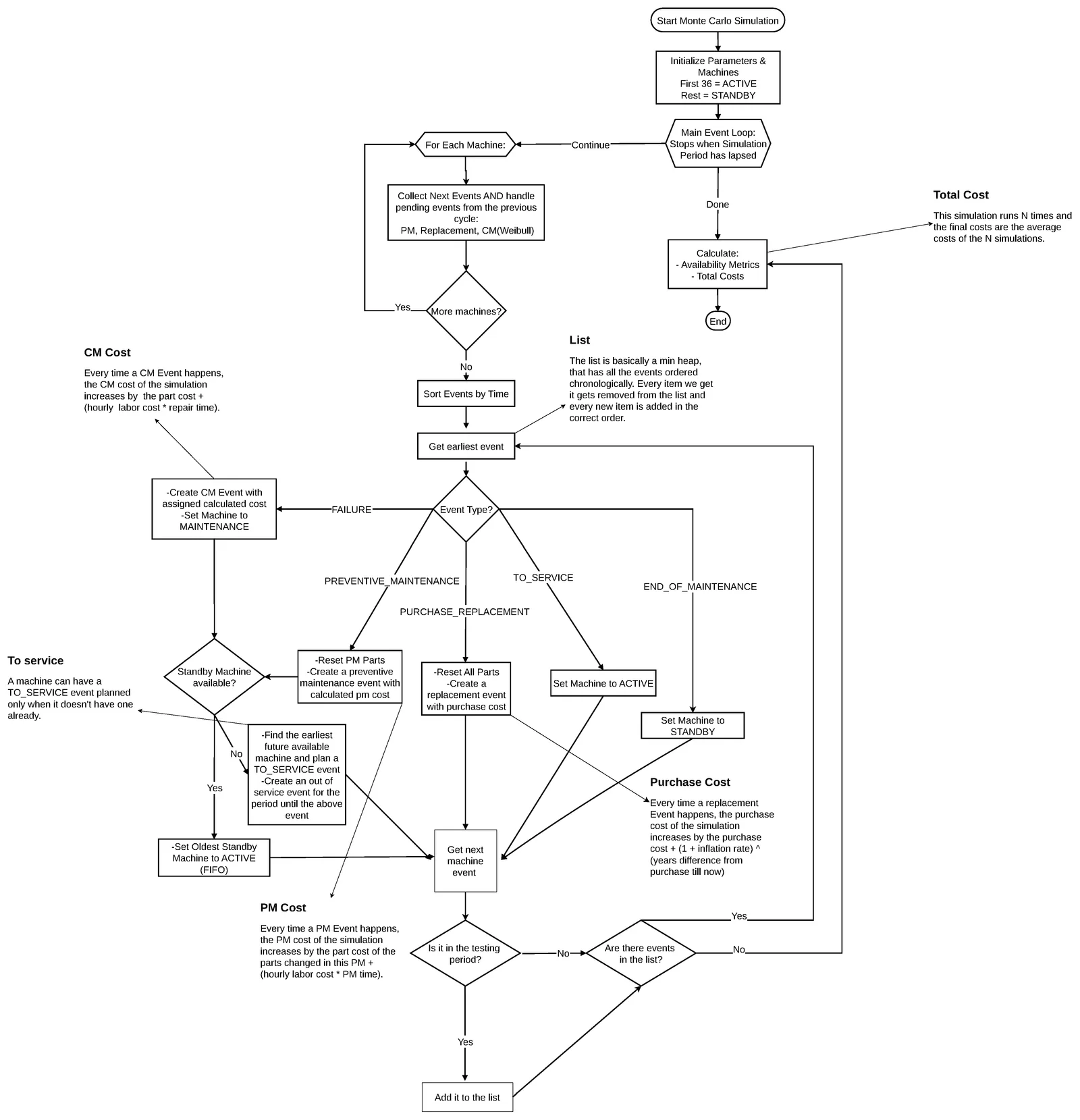
Simulation flowchart.

The defined events are monitored not only to calculate costs but also to estimate the service rate of the machines according to each alternative (Figure 4).

### 3.6. Simulation Workflow

#### 3.6.1. Step 1: Configure the Fleet

Before any alternative (Θ=(T,N)) is evaluated, the equipment, its costs, and its maintenance programme must exist. Each sub-step below uses the same inputs/outputs format as the rest of this section.

- Cost

Because a single cost profile can be shared across many machines or spare parts (for instance, all sensors might use the same cost data), it is usually one of the first things set up. Everything downstream (machines, spare parts, PM cycle years) links to a cost profile, which is described in Table 15.

- Machines

The information on inputs and outputs related to the machines is provided in Table 16. It corresponds to the equipment inventory: every machine in the fleet under study, regardless of which device family it belongs to.

- Spare Parts

The information on the workflow of spare parts is provided in Table 17.

- Preventive Maintenance Programme

The scope usually grows with each cycle year: year 1 might cover filters only; year 4 might add valves and sensors on top. The cycle’s total length is $I_{p}\;\times$ the number of cycle years. This step, described in Table 18, only defines what preventive maintenance costs and replaces; it does not yet say which machines actually follow the programme. That comes next, in step 1.5.

- Enrolment of Machines into the PM Programme

As shown in Table 19, the stagger index spreads each enrolled machine’s PM dates out relative to the rest of the fleet, so that the whole fleet does not undergo preventive maintenance all at once. It is assigned automatically rather than manually.

- Once steps 1.1 to 1.5 are done, every input parameter in Table 20 has a concrete source, and the fleet is ready to be swept across alternatives in step 2.

#### 3.6.2. Step 2: Define the Alternatives

Each alternative is the pair of decision variables already defined in the Nomenclature: the replacement period *T* and the fleet size *N*. An alternative is therefore the tuple

(26) Θ=(T,N),Θ∈A

#### 3.6.3. Step 3: Evaluate Each Alternative

As shown in Table 21, each alternative passes through an evaluation function E:A→(α,C,Q), which breaks down into three independent sub-models: an availability sub-model α, a cost sub-model *C* (reusing *C*, $C_{v}$, $C_{m}$, $C_{c}$, $C_{p}$), and a qualitative sub-model *Q*. Instantiating an alternative immediately runs *M* Monte Carlo replications through the simulation engine and averages the results into *C* and α, as shown in Table 22. *Q*, by contrast, is supplied by the analyst.

### 3.7. Convergence Assessment

Each alternative was simulated with 5000 independent replications. Convergence was assessed with the bootstrap method [43,44]: the 95% confidence interval of the mean of each output was obtained, and convergence was accepted when its half-width was below 0.02% of the mean. For all alternatives, the outputs met this criterion.

### 3.8. 95/95 Tolerance Interval

The 95/95 tolerance interval (95/95 TI) provides the interval in which, with a confidence of 95%, 95% of all possible outcomes will fall [45]. Following a non-parametric approach based on the percentiles of the Monte Carlo results, it is possible to obtain boundaries for the 95/95 TI. This approach best represents the actual variability of the results, independently of the number of simulation replications, provided that the number of replications is sufficiently large, a condition met by the 5000 replications [45].

### 3.9. Computational Implementation

The simulation was implemented in Python 3.13.5 using NumPy 2.3.5, SciPy 1.16.3, pandas 2.3.3, pyDecision 5.1.1, Matplotlib 3.10.7, Plotly 6.5.0. Each alternative was evaluated over M=5000 Monte Carlo replications for the 15-year planning horizon. The pseudo-random number generator was initialised with a fixed seed = 42 to ensure that all alternatives used the same random draws.

## 4. Results

### 4.1. Ownership Cost Analysis

The total ownership cost, including the purchase cost, the corrective maintenance cost, and the preventive maintenance cost, revealed significant variation across the decision alternatives (Table 23).

The total cost provided in this table is the mean over M=5000 Monte Carlo replications ± the half-width of the 95/95 TI. This half-width value is expressed in parentheses as a percentage of the mean. Because the purchase cost is set by each alternative and is not random, the 95/95 TI really reflects the variability of failures and corrective maintenance.

The replacement period mainly affects purchase cost, as it determines the frequency of fleet replacement over the study horizon. Purchase cost accounts for 88% of the total cost for the 7-year period and 72% for the 15-year period (38 units). The fleet size increases the cost within each replacement period: from 38 to 45 units, total cost increases by 22.4% for the 7-year period, 17.9% for the 9-year period, 23.6% for the 12-year period, and 17.1% for the 15-year period.

The total cost decreases as the replacement period gets longer: for 38 units, the cost is reduced by 15.6% from 7 to 9 years, by 21.9% from 9 to 12 years and by 26.9% from 12 to 15 years.

The EAC is also given in Table 23.

### 4.2. Service Rate

For simulation purposes, we define the number of available units in normal operation as the minimum of 36 (the number of ICU beds) and the number of devices available whenever an event changes the status of a device:

(27) $$N_{av}=\min\left(36,\;N_{use}+N_{sb}\right)$$

During a crisis, the number of available equipment is defined as the minimum of 44 (the maximum capacity of the hospital observed during COVID) and the number of devices available at the occurrence of a new event that changes the status of devices:

(28) $$N_{av}=\min\left(44,\;N_{use}+N_{sb}\right)$$

**Figure 5:**
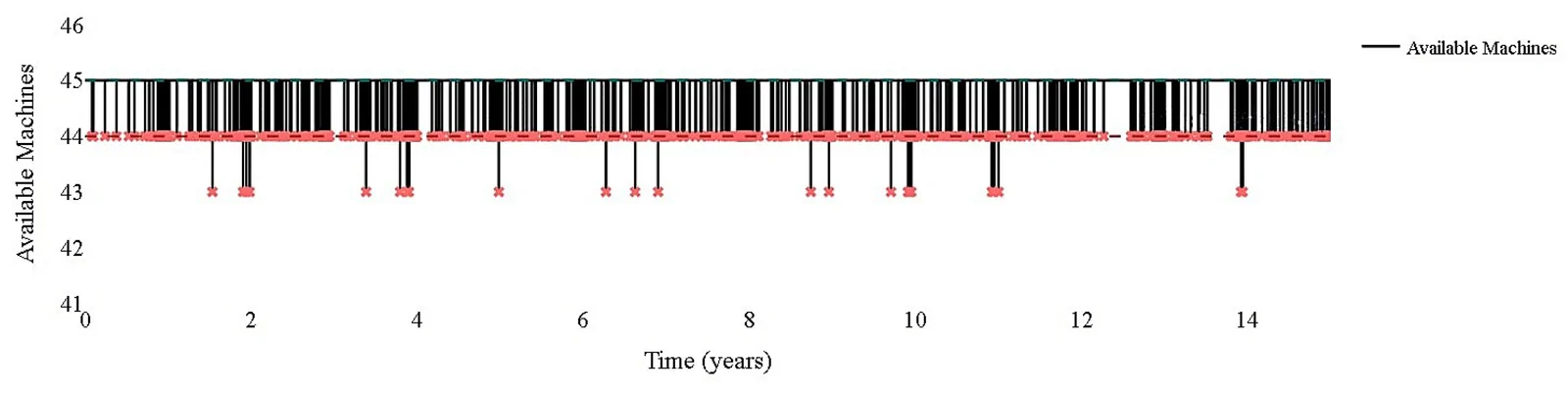
Service rate of ventilators for alternative 6 (N=45, T=9).

All the suggested alternatives achieved a 100% service rate under normal operating conditions (Figure 5), showing that even the smallest suggested fleet size provides the required capacity for daily ICU operations.

However, in a crisis, a threshold of 44 units of equipment was taken as the requirement for the hospital to operate at full capacity in the face of a surge in patients. The service rate in this simulation showed a strong relationship with fleet size, independent of the replacement period, resulting in three distinct service-rate levels for the equipment fleet (Table 24).

The difference between the largest and the smallest fleets amounts to six ventilators that are unavailable at the peak of a crisis for 38-unit alternatives compared to 45-unit ones. During emergency scenarios, this shortfall might have serious clinical implications. The effect of the fleet size on the service rate of equipment reflects that:

- Larger fleets cover for single equipment failures without reducing the operational capacity;
- A larger number of units enables simultaneous corrective and preventive maintenance without affecting the service rate;
- A larger fleet size ensures faster replacement of failed equipment, even during simultaneous failures.

### 4.3. Criteria Weights

Since all the suggested alternatives achieved a 100% service rate under normal operating conditions, in this application, it was decided to eliminate this criterion from the decision process, as it does not discriminate between the decision alternatives in this case.

The complete comparison matrix becomes:

(29) $$A=\left[\begin{array}{ccccc} 1 & 3 & 5 & 7 & 9\\ \frac{1}{3} & 1 & 3 & 5 & 7\\ \frac{1}{5} & \frac{1}{3} & 1 & 3 & 5\\ \frac{1}{7} & \frac{1}{5} & \frac{1}{3} & 1 & 3\\ \frac{1}{9} & \frac{1}{7} & \frac{1}{5} & \frac{1}{3} & 1 \end{array}\right]$$

Criterion 1 has strong importance over criterion 3 ($a_{13}=5$), and extreme importance over criterion 5 ($a_{15}=9$).

The results were as given in Table 25.

#### 4.3.1. Consistency Check

As presented in Table 26, since 0.0542<0.1, the consistency ratio is below the acceptable threshold, indicating consistent criteria judgments.

#### 4.3.2. Performance Score Table: Alternatives and Criteria

Table 27 represents the decision matrix, in which each alternative is assigned scores for its performance on each criterion.

### 4.4. Decision Results

Integrating the criteria weights and the alternatives’ scores produced the priority ranking of alternatives presented in Table 28. The decision scores represent priority values, where the higher the score, the better the overall performance of the alternative with respect to the evaluation criteria.

Alternative 6 (9-year replacement period, 45-ventilator fleet) was the best-ranked among the evaluated fleet replacement strategies, with the highest priority score (0.8657). This alternative achieves perfect performance across qualitative criteria (staff satisfaction: 10, sensitivity to obsolescence: 10, ease of procurement and logistics: 10) and ensures 100% service rate of equipment in both normal and crisis situations. In fact, alternative 6 does not offer the lowest total cost; it represents the optimal cost value to balance the other criteria scores.

Alternative 12 (15-year replacement period, 45-ventilator fleet) had a priority score of 0.8523 and was ranked second. This alternative shows that extended ventilator life remains viable when cost constraints dominate. Alternative 12 guarantees a 100% service rate of equipment in both normal and crisis situations but at a cost almost 43% lower than that of alternative 6, in exchange for the lowest scores in staff satisfaction and sensitivity to obsolescence and a reduced logistics score. However, the performance of alternative 12 appears to be linked to the horizon, as the EAC analysis shows. Under steady-state operation, and using the EAC instead of the 15-years cost, alternative 9 would be ranked second (the cost difference is reduced to 31% when examining EAC).

The 9-year alternatives scored 10 on all qualitative criteria while offering mid-range costs and crisis service rates that scale with total fleet size.

The 15-year alternatives received the lowest scores for staff satisfaction and technology features, but, due to lower replacement frequency, the logistics score was moderately good, and the total cost was remarkably low.

All the 7-year alternatives rank in the bottom positions: 10th, 11th, and 12th. This performance mainly reflects the impact of the total cost criterion, on which the cost of these alternatives is almost double that of the least-cost option. The 7-year replacement period alternatives consume budget resources due to frequent fleet replacements, while showing moderately good performance on qualitative criteria. Each 7-year alternative is dominated by the 9-year alternative of the same fleet size, which has a lower cost, the same crisis service rate and equal or higher qualitative scores.

#### 4.4.1. Critical Role of Crisis Service Rate

**Figure 6:**
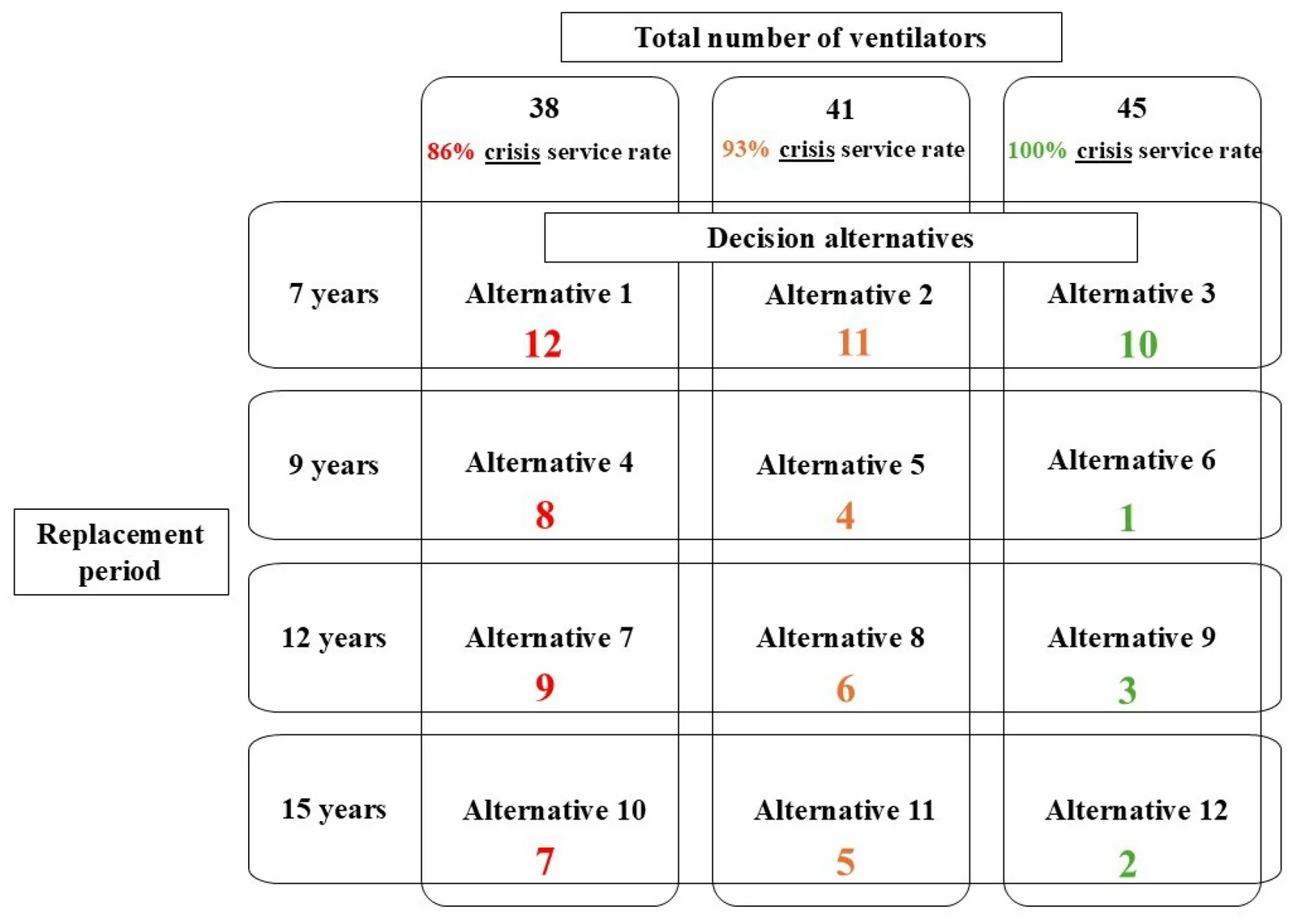
Classification of alternatives.

The total fleet size has a major impact on the service rate during crises. In each replacement period cluster, the ranking follows the fleet size. For example, for a 9-year replacement period, alternatives 6 (N=45), 5 (N=41), and 4 (N=38) ranked 1st, 4th, and 8th (Figure 6). The relationship between fleet size and crisis service rate derives mainly from reliability modelling, where larger fleets provide greater access to equipment: more units for immediate replacement if a failure occurs and greater maintenance capacity to support service rotation.

#### 4.4.2. Evaluating on an Annual-Cost Basis

Even when the EAC is used (Table 23) instead of the 15-year cost, alternative 6 remains first (0.891), followed by alternatives 9 (0.877) and 5 (0.871); alternative 12 falls to fifth place (0.850). The 15-year cost favours the 15-year alternatives because their replacement age equals the horizon: the units bought at t = 0 are due for replacement exactly at H, which is not charged. On an annual basis, alternative 12 costs 31% less than alternative 6, compared with 43% over the 15-year horizon.

### 4.5. Sensitivity Analysis

To determine criteria weights using the AHP method, it is necessary to introduce subjective expert judgment into the process. This adds uncertainty to the decision-making framework; therefore, sensitivity analysis is essential for assessing the robustness of the alternatives’ ranking and determining whether the preferred decision alternative remains stable across defined variations in criteria weights. This step is also necessary to identify critical weight thresholds, evaluate how different perspectives affect decision outcomes, and support the decision process by demonstrating the logic and expertise behind the weight assignment.

The final priorities of alternatives are highly dependent on the weights attached to the criteria [46]. Therefore, to assess the robustness of the ranking, a one-at-a-time (OAT) sensitivity analysis was conducted on each criterion weight. This method systematically varies each criterion weight individually while proportionally adjusting the rest of the weights to keep the sum of weights to 1.

The weights of Table 25 are derived from the pairwise judgments of Equation (29), which are the actual inputs of the decision makers. Robustness is therefore assessed in two steps.

First, each weight is varied one at a time, the other weights being rescaled proportionally, and the exact value $w_{k}^{*}$ at which alternative 6 loses the first rank is computed. Since the scores are linear in the weights, $w_{k}^{*}$ is the intersection of two straight lines (Table 29). Second, each of the ten judgments is moved one step up or down on the Saaty scale (resulting in 19 matrices, all with CR≤0.07), and the weights and ranking are recomputed. Alternative 6 remains first in all 19 cases.

The qualitative scores are tested in the same way. Raising or lowering one score by one grade for one replacement-age group changes the first-ranked alternative in 5 of 19 cases (Table 30). Alternative 12 overtakes when the 15-year fleets are rated higher or the 9-year obsolescence is lower. Alternative 9 overtakes when the 9- or 12-year staff-satisfaction scores change. Under the equivalent annual cost (Section 4.1), only alternative 9 can displace alternative 6. These scores therefore deserve the most attention when the method is applied.

Two alternatives are considered tied when their scores differ by less than $\varepsilon =10^{-9}$. Tied alternatives receive the average rank of the positions they share, instead of an arbitrary order. Ties arise mainly at the endpoint $w_{i}=1$, where the model reduces to a single criterion evaluated on the qualitative scale. At this point, alternatives with the same fleet size tie on the crisis service rate; the 7- and 9-year alternatives tie on sensitivity to obsolescence; the 9- and 12-year alternatives tie on ease of procurement and logistics; and alternatives with equal staff satisfaction scores tie within their replacement period groups.

A rank change is considered decision-relevant only if it affects the three top-ranked alternatives and occurs within the weight region compatible with the experts’ ordinal comparison of the criteria used to build the AHP pairwise matrix. Reversals outside this region are reported for transparency but do not modify the recommendation. When two alternatives exchange the first rank in this region, they are reported as a set of competing alternatives, and the decision maker is responsible for choosing a strategy from the set of recommendations.

### 4.6. Cost Variability

The total cost of each alternative varies between simulated futures because failures are random. The 95/95 tolerance intervals in Table 23 have half-widths between 0.22% and 0.52% of the mean, since the purchase cost, which is the largest share of the total, is fixed by the alternative.

To test whether this variability can change the preferred alternative, we evaluated the least favourable combination for alternative 6: its cost at the upper bound of its tolerance interval and every other alternative’s cost at its lower bound. Alternative 6 remains first. The 95/95 TI would have to be about 15 times wider to change the first rank.

Furthermore, a common change in the price level would not affect the ranking, because the cost scores as given in Equation (8) are normalised and thus not sensitive to a common scale factor. The yearly price revision, set by framework contracts, would have to exceed about 6% for alternative 6 to lose the first rank.

Parametric uncertainty in cost inputs remains the main risk for the decision and should be considered in future studies.

## 5. Discussion

In this study, a hybrid AHP-weighted-sum MCDM method was used to evaluate 12 equipment management alternatives that combine fleet size and fleet replacement period. This analysis produced several findings that confirm existing medical equipment management standards but challenge some real-world application patterns. L. Fennigkoh [47] suggested that medical equipment replacement should balance clinical reliability, equipment service and support, and cost benefits. Although he did not use the same method, he recognised that an optimal replacement strategy requires MCDM rather than single-factor evaluation. Taghipour et al. [30] proved that age-based replacement of medical equipment can achieve cost-effectiveness when the time of replacement balances reliability and expenses. Their study’s findings for optimal replacement intervals are consistent with the 9–12-year replacement period recommended here for the replacement of ventilators.

On a technological level, R.L. Chatburn and E. Mireles-Cabodevila [48] evaluated the evolution of mechanical ventilation technology and concluded that innovations are incremental, not revolutionary: optimised user interfaces or enhanced monitoring, but no changes to the therapeutic mechanism. This supports the fact that a 9- or 12-year-old ventilator can remain clinically reliable and that it is not necessary to replace ventilators every 7 years for new features.

### 5.1. Crisis Capacity

This research showed that fleet size determines the service rate during a crisis. This is also a lesson from the COVID-19 pandemic, which revealed ventilator shortfalls globally. Wang et al. [49] argued that crisis preparedness requires stockpiling equipment rather than acquiring it on demand. Healthcare institutions should always provide extra capacity for crises such as pandemics or mass-casualty events. Ranney et al. [1] documented the shortage of ventilators and consumables, with demand exceeding supply during COVID-19 because of disruptions in the supply chain and the lack of preparedness of healthcare facilities for the pandemic, which led to desperate measures such as reusing disposable equipment; lifting strict standards on medical products to cover the shortages; and urging production despite high demand for raw materials, which makes new equipment more expensive, when available. Our model’s recommendation of 45-unit fleets aligns with this principle as an essential crisis resilience strategy. Additional available ventilators provide life support for more patients and help avoid potential tragedies.

### 5.2. MCDM in Healthcare Asset Management

MCDM methods are a useful decision tool in most applications. The healthcare sector remains a sensitive, high-risk environment that requires human expertise to evaluate the situation and decision options; therefore, in most healthcare facilities, decisions are still made based on the clinical and financial expertise of decision makers. Given the various factors that affect the decision-making process, choosing an acceptable option remains challenging, as decision makers are always influenced by their judgment and the field of expertise they represent (medical, financial, technical, or logistical). Several studies have addressed the use of MCDM methods in healthcare and medical equipment management, proving their usefulness in effective decision making. Liberatore and Nydick provided a comprehensive review of AHP application in healthcare for medical equipment assessment and selection [50]. Sloane documented several examples of AHP medical applications [51], such as selecting medical residents, selecting neonatal ventilators, assisting surgical decisions, and improving patient discharge planning. However, most previous studies focused on initial equipment selection, equipment classification, or medical treatment rather than equipment lifecycle management strategies. This study addresses this gap by using MCDM methods to help define replacement and fleet sizing strategies, showing the worth of this methodology for strategic planning and asset management beyond the pre-procurement selection. The second dimension of this study is the inclusion of crisis resilience as a factor for replacement planning. While crises in medical fields are often addressed from a clinical perspective, this study contributes to the hospital’s preparedness by ensuring the availability of a reliable fleet of life-support equipment, specifically ICU ventilators.

### 5.3. Limitations and Future Research Directions

This research has limitations that require acknowledgment. First, the reliability analysis assumes that failures follow Weibull distributions with defined parameters; however, several factors introduce uncertainty: maintenance quality, intensity of use, environmental working conditions, technology evolution, and different equipment manufacturers. Ventilator-level ageing is not modelled: parts are renewed as good as new, so failure frequency does not grow with ventilator age. Replacement age therefore acts on cost and on the qualitative criteria, not on availability. Likewise, because repairs take only a few hours, simultaneous ventilator stoppages are rare, so the crisis service rate is strongly linked to the fleet size.

Compliance with manufacturer recommendations is challenging, as the selected replacement period may not align with the manufacturer’s replacement and maintenance contracts or standards. This analysis assumes that the equipment remains compliant with health regulations and standards throughout the recommended replacement periods.

While using an MCDM method adds an objective dimension to the process, the AHP model for weight determination still relies on expert judgment that can be subjective.

The ranking applies to an additive value model. Methods based on other preference models, such as distance to an ideal solution (TOPSIS) or outranking (PROMETHEE), treat compensation between criteria differently and can rank the alternatives differently. The choice of aggregation model is therefore part of the decision maker’s preference structure, not a neutral computational step.

Future research needs to focus on using MCDM methods for medical asset management of other critical equipment that provides life support, requires high capital investment, and involves multiple criteria, such as dialysis machines, anaesthesia equipment, and medical imaging equipment.

Future applications should make the preference model explicit when choosing the aggregation method, since compensatory, distance-based and outranking methods can lead to different recommendations from the same data.

A further extension is to treat cost and reliability parameters as random variables and carry that uncertainty through the alternatives’ scores, so alternatives can be compared through their score distributions instead of single values. Such an approach would allow statistically grounded statements about alternatives with close scores. Likewise, the cost model uses a finite 15-year horizon with nominal, undiscounted costs, and it does not credit the value of the fleet remaining at the horizon. A residual value would require a depreciation profile for the ventilators, which depends on the hospital’s accounting rules rather than on the model. This is why the horizon model favours the replacement age equal to the horizon length. The EAC removes this effect but assumes a steady-state cycle and ignores the transition from the current fleet. In the present case, alternative 6 ranks first under both conventions, although the second-best depends on the convention.

Furthermore, the cost variability reported here comes from random failures only. Weibull parameters, repair times, labour rates and unit prices are fixed inputs, and their uncertainty is not propagated through the model.

Along the same lines, predictive classification of devices by expected time to failure [52] could replace the fixed Weibull parameters used here with estimates learned from the maintenance history of an equipment fleet.

The crisis resilience representation adopted here can be extended in two directions. First, the crisis requirement was expressed as a single capacity threshold corresponding to the maximum capacity that the hospital’s human resources can absorb. Testing a range of demand levels would show how the ranking of alternatives evolves with crisis severity.

Second, richer readiness indicators, such as the probability of having at least the required number of functional devices, the expected unmet demand and the associated shortage duration, would characterise the behaviour of the fleet under a prolonged surge and could be the subject of future work.

It would be interesting to simulate coincident crises. Delayed repair conditions and spare-parts shortages constitute a logistics or supply-chain crisis, whose interaction with a demand-surge crisis would require a model of multiple simultaneous crisis events.

A methodology is needed to integrate research results and MCDM applications with healthcare facilities’ asset management systems. Transitioning from current practices to a new tool can be challenging and requires guidelines and support to help hospitals adopt this decision system. Such support could focus on integrating Computerised Maintenance Management Systems (CMMS) and management platforms, helping hospitals identify and evaluate critical criteria and designing staff training programmes.

## 6. Conclusions

An illustrative case study was presented to demonstrate the proposed methodology: Integrating criteria weights and alternatives’ performance scores into the decision model ranked 12 alternatives according to criteria directly related to the decision problem.

For this illustrative case study, alternative 6 (9-year replacement period, 45-unit fleet) was selected as the best-ranked fleet management strategy among the alternatives. It reaches the highest qualitative scores and a 100% service rate in normal and crisis situations at a moderate cost, and it remains first under any single one-step change of the pairwise judgments. Its nearest challenger depends on how cost is assessed: alternative 12 over the 15-year horizon, alternative 9 on an annual basis. A one-grade change in the qualitative scores of these alternatives can reverse the first rank.

The ranking is objective with respect to the criteria and weights entered into the model. The results depend heavily on the inserted factors, which are the decision maker’s input, although they are not the only factors in the decision.

The 12 alternatives compared in the study do not constitute an exhaustive exploration of decision strategies for medical equipment. The replacement periods were bounded by the manufacturer’s and the World Health Organization’s recommendations, while the fleet sizes correspond to the fleet currently operated by the hospital. The alternative presented as the preferred decision is considered the best-ranked among those the hospital can realistically implement, but not a global optimum. A continuous search over replacement ages and fleet sizes may reveal intermediate strategies and extend this work.

MCDM methods serve as guidance and recommendation tools for decision making, but the decision maker does not need to follow the results exactly or choose the optimal suggested alternative. The decision maker can follow the model’s recommendation and adjust it based on available resources, the environment, limitations, risk assessments, and budgeting. MCDM tools value human input and expert judgment in evaluating alternatives’ performance, provide an objective ranking and, by design, leave the final decision to the decision maker. The choice of the optimal alternative by the decision maker might depend on:

- The compromise interval the hospital is willing to accept regarding each criterion;
- The risk the hospital is willing to take regarding crisis resilience;
- Budget planning and the effect of investing in this equipment on other resource allocations.

## Figures and Tables

**Table 1:** Maintenance strategies according to the criticality level.

Criticality Level	Maintenance Strategy
High	Proactive, predictive, time-based maintenance
Medium	Proactive or time-based maintenance
Low	Corrective maintenance

**Table 2:** Saaty scale of level of importance for AHP criteria comparison.

Intensity of Importance	Verbal Judgment
1	Equal importance
3	Moderate importance of one over the other
5	Essential or strong importance
7	Very strong importance
9	Extreme importance
2, 4, 6, 8	Intermediate values between 2 adjacent judgments

**Table 3:** The ICU ventilator fleet.

Total Number of Ventilators (*N*)	Number of Ventilators in Service ($N_{\mathrm{use}}$)	Number of Ventilators on Standby ($N_{\mathrm{sb}}$)
45	36	9

**Table 4:** The initial ages of the ICU ventilators.

Number of Units	Age *a* (Years)
25	15
20	6

**Table 5:** The ICU ventilator fleet during COVID-19.

Total Number of Ventilators ($N_{\mathrm{crisis}}$)	Number of Ventilators in Service ($N_{{\mathrm{crisis}}_{\mathrm{use}}}$)	Number of Ventilators on Standby ($N_{{\mathrm{crisis}}_{\mathrm{sb}}}$)
60	44	16

**Table 6:** Decision alternatives for managing ICU ventilators in terms of replacement period and the number of units to keep in stock.

Alternative	Replacement Age *T* (Years)	Number of Ventilators on Standby ($N_{\mathrm{sb}}$)	Total Number of Units $N=N_{\mathrm{use}}+N_{\mathrm{sb}}$
1	7	2	38
2	7	5	41
3	7	9	45
4	9	2	38
5	9	5	41
6	9	9	45
7	12	2	38
8	12	5	41
9	12	9	45
10	15	2	38
11	15	5	41
12	15	9	45

**Table 7:** Score description table of the staff satisfaction criterion.

Description	Score	Evaluation
The equipment fleet is homogeneous, but it does not meet staff preferences for models (old or new), and is hard to use, handle and maintain.	2.5	Poor
The equipment fleet is homogeneous, moderately user-friendly, and maintainable, but does not align with the staff’s preferences for optimal models.	5	Average
The equipment aligns with staff preferences (either older models or requested newer models), the fleet is homogeneous, and it is moderately easy to use and handle, with a minor learning curve or inconveniences for some staff.	7.5	Good
The equipment fully aligns with staff preferences (optimal mix of reliable models or the latest requested ones), the fleet is homogeneous, and is easy for all staff to use, handle, and maintain.	10	Excellent

**Table 8:** Score description table of the sensitivity to obsolescence criterion.

Description	Score	Evaluation
The equipment is outdated and has obsolete features that delay the patient’s treatment and cause major difficulties for the user.	0	Unacceptable
The equipment has basic features for effective treatment but causes discomfort for both the patient and the user and interruptions or delays in treatment.	2.5	Poor
The equipment has adequate clinical features for effective treatment with some modern interface elements, but lacks advanced options, requires significant user effort, and causes some patient discomfort.	5	Average
The equipment is up to date with modern clinical features and a good user interface, enabling effective patient treatment, but it may require moderate user effort and training.	7.5	Good
The equipment is up to date and has the most modern clinical features and an optimal user interface for effective patient treatment and comfort, and requires minimal user effort.	10	Excellent

**Table 9:** Score description table of the ease of procurement and logistics criterion.

Description	Score	Evaluation
The fleet has equipment renewed at variable replacement intervals with multiple suppliers, inconsistent maintenance plans for multiple subgroups of the equipment fleet, complex maintenance actions, and moving and storage logistics.	0	Unacceptable
The fleet has equipment renewed at multiple fixed replacement intervals with multiple suppliers, different maintenance plans for multiple subgroups of the equipment fleet, causing multi-level maintenance actions, and moving and storage logistics.	2.5	Poor
The fleet has equipment renewed at fixed replacement intervals under the same purchase contract with the supplier, with different maintenance management plans across the equipment fleet, including maintenance actions, moving, and storage.	5	Average
The equipment fleet is renewable at a fixed replacement interval with a single supplier but variable maintenance management plans across fleet subgroups, causing moderate complexity in maintenance actions, moving, and storage logistics.	7.5	Good
The equipment fleet is renewed at consistent and shorter intervals, with a single supplier and a fixed maintenance management plan for the equipment fleet, including clear maintenance actions and moving and storage processes.	10	Excellent

**Table 10:** Preventive maintenance: 6-year cycle.

Year	Duration (h)	Hourly Labour Rate (€/h)
1	1	60
2	2	60
3	2.5	60
4	3	60
5	4	60
6	5	60

**Table 11:** Sensor and battery costs and downtime.

Part	Part Cost $c_{\mathrm{sp}}$ (EUR)	Downtime (h)	Hourly Labour Rate (EUR/h)
Sensor	70	4	60
Battery	260	5	60

**Table 12:** ICU ventilator statuses.

Equipment Status	Description
Active	In service and connected to an ICU bed
Standby	Available and ready for use
In maintenance	Not available due to a failure or preventive maintenance

**Table 13:** The ICU ventilator events.

Event	Description
Failure	Failure detected; equipment sent to corrective maintenance and replaced by available equipment, if any exists (equivalent to the “To service” event)
Back to service	An equipment replacement from the standby stock, or after the end of maintenance if no stock is available
End of maintenance	End of corrective maintenance and return to stock, or to service (equivalent to the “Back to service” event if equipment is needed in service)
Preventive maintenance	Start of preventive maintenance if there are no failures (in the scheduled period for PM)
Replacement	End of replacement period for the machine; decommissioning and replacement with a new one

**Table 14:** Parts Weibull parameters.

Part	β	η (Years)
Sensor	1.8	2.25
Battery	1.8	3.37

**Table 15:** Cost profile: inputs and outputs.

Inputs	*c_h_* Hourly labour cost Corrective-maintenance hours *T_c_* Corrective-maintenance MTTR hours: the average time to fully restore a machine to service after a failure, including logistics and waiting time on top of hands-on repair labour (always ≥ corrective-maintenance hours, which count only the hands-on repair time) *T_p_* Preventive-maintenance hours
Outputs	Maintenance cost data for a part or machine, reusable across many machines and spare parts

**Table 16:** Machines: inputs and outputs.

Inputs	Machine name, code, purchase year and *c_v_* purchase cost Machine-level Weibull parameters (*β*, *η*) Cost profile (step 1.1)
Outputs	One machine in the fleet

**Table 17:** Spare parts: inputs and outputs.

Inputs	Spare-part type and its own Weibull parameters (*β*, *η*) The machine it belongs to A cost profile (either shared with the machine’s own profile or a dedicated one)
Outputs	One spare part per replaceable component (e.g., battery, sensor, valve, gasket, filter, bearing, manufacturer kit, or any other part relevant to the device family under study)

**Table 18:** Preventive maintenance programme: inputs and outputs.

Inputs	*I_p_* Interval between visits (e.g., 1 year) A cost profile for each year of the PM cycle The list of spare-part types replaced that year
Outputs	One preventive maintenance programme with cycle years and the parts to be replaced in each year of the cycle

**Table 19:** Enrolment of machines into the preventive maintenance programme: inputs and outputs.

Inputs	A machine A PM programme
Outputs	The machine’s preventive maintenance programme is set. A stagger index is also assigned, as described below.

**Table 20:** Definition of the alternatives: inputs and outputs.

Inputs	*N* Candidate fleet sizes and *T* replacement periods to compare *H* Simulation horizon *M* Monte Carlo replication count $N_{need}$, $N_{crisis}$—Normal and crisis thresholds *Q* Analyst-supplied qualitative scores The AHP pairwise comparison matrix *A*
Outputs	One simulation profile S (a reusable bundle of the above parameters). One alternative Θ=(T,N) per fleet-size × replacement period combination in the sweep.

**Table 21:** Evaluation of an alternative: inputs and outputs.

Inputs	A single alternative Θ∈A, or the full alternative space A The simulation profile S (Table 20)
Outputs	α (normal and crisis availability), *C* (total cost), and *Q* (carried through unchanged) per alternative. The full set of simulation events *E* generated per simulation.

**Table 22:** Availability sub-model: inputs and outputs.

Inputs	Θ=(T,N) β, η per machine and per spare part *H* $N_{need}$, $N_{crisis}$
Outputs	Normal situation α and crisis situation α, each a real number in [0,1]

**Table 23:** Ownership cost for each replacement period and fleet size (ventilator units in the system).

T (Years)	Fleet Size	Purchase Cost (€)	Total Cost (€)	Average Cost per Unit (€)	EAC (€/Year)
7 years	38	2,656,508	3,012,820 ± 7595 (0.25%)	79,285	159,468
	41	2,916,615	3,301,766 ± 7630 (0.23%)	80,531	172,105
	45	3,263,425	3,687,049 ± 8115 (0.22%)	81,934	188,956
9 years	38	2,152,535	2,543,470 ± 7460 (0.29%)	66,933	131,618
	41	2,317,163	2,738,947 ± 7670 (0.28%)	66,804	142,008
	45	2,536,668	2,999,849 ± 7945 (0.26%)	66,663	155,879
12 years	38	1,583,788	1,985,353 ± 7510 (0.38%)	52,246	105,938
	41	1,753,903	2,186,535 ± 7985 (0.37%)	53,330	114,259
	45	1,980,722	2,454,831 ± 8110 (0.33%)	54,552	125,357
15 years	38	1,047,557	1,451,999 ± 7505 (0.52%)	38,210	90,296
	41	1,122,555	1,558,403 ± 7905 (0.51%)	38,010	97,390
	45	1,222,553	1,700,114 ± 8265 (0.49%)	37,780	106,837

**Table 24:** Crisis service rate by fleet size.

Fleet Size	Crisis Service Rate (%)
45 units	100.00
41 units	93.11–93.12
38 units	86.29–86.30

**Table 25:** The criteria weights.

Criterion	Weight
Service rate in crisis	0.5028
Total cost of ownership	0.2602
Staff satisfaction	0.1344
Sensitivity to obsolescence	0.0678
Ease of procurement and logistics	0.0348

**Table 26:** Consistency ratio.

Metric	Result
$\lambda_{max}$	5.2426
Random Index RI	1.12
Consistency ratio	0.0542

**Table 27:** Performance scores table.

Alternative	Service Rate in Crisis (%)	Total Cost (€)	Staff Satisfaction	Sensitivity to Obsolescence	Ease of Procurement and Logistics
1	86.300	3,012,820 ± 7595 (0.25%)	7.5	10	7.5
2	93.120	3,301,766 ± 7630 (0.23%)	7.5	10	7.5
3	100.000	3,687,049 ± 8115 (0.22%)	7.5	10	7.5
4	86.300	2,543,470 ± 7460 (0.29%)	10	10	10
5	93.120	2,738,947 ± 7670 (0.28%)	10	10	10
6	100.000	2,999,849 ± 7945 (0.26%)	10	10	10
7	86.300	1,985,353 ± 7510 (0.38%)	7.5	7.5	10
8	93.120	2,186,535 ± 7985 (0.37%)	7.5	7.5	10
9	100.000	2,454,831 ± 8110 (0.33%)	7.5	7.5	10
10	86.290	1,451,999 ± 7505 (0.52%)	5	5	7.5
11	93.110	1,558,403 ± 7905 (0.51%)	5	5	7.5
12	100.000	1,700,114 ± 8265 (0.49%)	5	5	7.5

**Table 28:** Alternatives ranking.

Alternative	Rank	Score
Alternative 6 (9, 45)	1	0.8657
Alternative 12 (15, 45)	2	0.8523
Alternative 9 (12, 45)	3	0.8432
Alternative 5 (9, 41)	4	0.8431
Alternative 11 (15, 41)	5	0.8378
Alternative 8 (12, 41)	6	0.8274
Alternative 10 (15, 38)	7	0.8213
Alternative 4 (9, 38)	8	0.8195
Alternative 7 (12, 38)	9	0.8107
Alternative 3 (7, 45)	10	0.8000
Alternative 2 (7, 41)	11	0.7773
Alternative 1 (7, 38)	12	0.7540

**Table 29:** Sensitivity of the criteria weights.

Criterion	Baseline Weight	Threshold, Total Cost	Threshold, EAC	Weight Range, One-Step Judgment Changes
Crisis service rate	0.503	0.264 (A10 below)	0.301 (A5 below)	0.474–0.521
Total cost	0.260	0.286 (A12 above)	0.326 (A9 above)	0.243–0.286
Staff satisfaction	0.134	0.110 (A12 below)	0.084 (A9 below)	0.123–0.144
Obsolescence	0.068	0.042 (A12 below)	0.013 (A9 below)	0.060–0.075
Procurement and logistics	0.035	none	none	0.034–0.037

**Table 30:** Leading alternative under one-step score changes.

Score Change	Leader, 15-Year Cost	Leader, Annual Cost
Staff satisfaction, T= 9 years: 10 → 7.5	A9	A9
Staff satisfaction, T= 12 years: 7.5 → 10	A9	A9
Staff satisfaction, T= 15 years: 5 → 7.5	A12	A6
Obsolescence, T= 9 years: 10 → 7.5	A12	A9
Obsolescence, T= 12 years: 7.5 → 10	A6	A9
Obsolescence, T= 15 years: 5 → 7.5	A12	A6
All other one-step changes	A6	A6

## Data Availability

The original contributions presented in this study are included in the article. The code is available at the GitHub repository at https://github.com/MolkSouguir/MCDM-Simulation-for-ventilators-fleet (accessed on 26 October 2026). Further inquiries can be directed to the corresponding author.
